# Mechanism of Action of Gepotidacin: Well-Balanced Dual-Targeting against *Neisseria gonorrhoeae* Gyrase and Topoisomerase IV in Cells and In Vitro

**DOI:** 10.1021/acsinfecdis.5c00497

**Published:** 2025-10-09

**Authors:** Alexandria A. Oviatt, Jessica A. Collins, Chelsea A. Mann, Jianzhong Huang, Karen Mattern, Pan F. Chan, Neil Osheroff

**Affiliations:** † Department of Biochemistry, 12327 Vanderbilt University School of Medicine, Nashville, Tennessee 37232, United States; ‡ Infectious Diseases Research, 33139 GSK, Collegeville, Pennsylvania 19426, United States; § Infectious Diseases Research, Vaccines Research and Development Infectious Diseases Research, 33139 GSK, Collegeville, Pennsylvania 19426, United States; ∥ Departments of Biochemistry and Medicine (Hematology/Oncology), 12327 Vanderbilt University School of Medicine, Nashville, Tennessee 37232, United States

**Keywords:** gepotidacin, gyrase, topoisomerase IV, *Neisseria gonorrhoeae*, DNA cleavage, DNA supercoiling/decatenation

## Abstract

Gonorrhea, a sexually transmitted infection caused by *Neisseria gonorrhoeae*, remains a major public health concern. Fluoroquinolones, which target gyrase and topoisomerase IV, once served as first-line therapy for gonorrhea. However, rising target-mediated resistance led to their removal from treatment guidelines. In response to growing antibacterial resistance, gepotidacin, a first-in-class triazaacenaphthylene, offers a promising new treatment strategy. Gepotidacin targets gyrase and topoisomerase IV but is structurally and mechanistically different from fluoroquinolones. A phase III clinical trial of gepotidacin in the treatment of uncomplicated urogenital gonorrhea demonstrated a positive outcome. However, interactions of the drug with *N. gonorrhoeae* gyrase and topoisomerase IV have not been reported. Consequently, we determined the targeting of gepotidacin in *N. gonorrhoeae* cells and its effects on purified *N. gonorrhoeae* gyrase and topoisomerase IV. Although fluoroquinolones primarily target gyrase in Gram-negative bacteria, gepotidacin displayed well-balanced dual-targeting of both gyrase and topoisomerase IV in cultured cells. Reduced gepotidacin susceptibility required concurrent target-specific mutations in both enzymes, predicting a low propensity for developing target-mediated resistance. Consistent with this cellular dual-targeting, gepotidacin inhibited gyrase-catalyzed DNA supercoiling and topoisomerase IV-catalyzed DNA decatenation at similar low micromolar concentrations. Gepotidacin also induced primarily single-stranded DNA breaks mediated by both enzymes at comparable concentrations. Finally, mutations in aspartic acid residues predicted to mediate important gepotidacin-protein interactions in *N. gonorrhoeae* gyrase (GyrA^D90^) and topoisomerase IV (ParC^D86^) markedly diminished the activity of gepotidacin against the respective enzymes. Our findings differentiate gepotidacin targeting and mechanism from those of fluoroquinolones and highlight its potential to combat drug-resistant gonorrhea.

## Graphical abstract

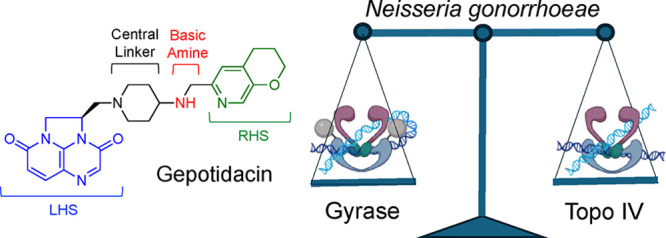

Gonorrhea is a sexually transmitted infection that is caused by the Gram-negative bacterium *Neisseria gonorrhoeae*.^1^ It infects the mucosal epithelium of the genitals, rectum, and throat in humans,^2,3^ and is one of the major causes of pelvic inflammatory disease.^2,4^ If left untreated, gonorrhea can lead to infertility and potentially death.^2,4^ It is the second most common sexually transmitted infection caused by a bacteria,^2^ eclipsed only by chlamydia, and in 2020 affected more than 82 million people worldwide.^2,5^ Drug-resistant *N. gonorrhoeae* is listed by the World Health Organization as a high priority pathogen^6^ that has the potential to join HIV/AIDs and herpes as the third incurable sexually transmitted disease.^7^ Furthermore, the Centers for Disease Control and Prevention (CDC) has categorized gonorrhea as an “urgent antibiotic resistance threat.”^8^

In 1993, the fluoroquinolone ciprofloxacin was approved as a frontline therapy against gonorrhea.^9^ However, in 2006, in response to increasing rates of fluoroquinolone resistance, the CDC removed ciprofloxacin from gonorrhea treatment guidelines.^10^ The most important mechanism that underlies fluoroquinolone resistance results from point mutations in specific amino acid residues of gyrase and topoisomerase IV, which are the targets for this antibacterial class.^11–14^ Gyrase and topoisomerase IV are homologous enzymes that fulfill pivotal roles in controlling DNA over- and underwinding and removing tangles and knots from the bacterial chromosome, respectively, by generating transient double-stranded breaks in the genetic material.^13–18^ To preserve genomic integrity during DNA cleavage, gyrase and topoisomerase IV form covalent linkages between their active-site tyrosine residues and the newly generated 5′-terminal phosphates of the cleaved double helix.^12–16^ This covalent enzyme-cleaved DNA complex, which is the target for fluoroquinolones, is known as the cleavage complex.^12–16^

Currently, approximately one-third of *N. gonorrhoeae* clinical isolates in the United States exhibit resistance to ciprofloxacin.^19,20^ In many regions of the world, especially Asia, fluoroquinolone resistance exceeds 90%.^20^ The current standard of care for gonorrhea is a combination of intramuscularly injected ceftriaxone and oral azithromycin.^21^ However, this regimen is becoming an increasing concern due to the rise in resistance to both antibiotics.^20^ Consequently, there is an urgent need to develop new, especially oral, antibacterials to treat gonorrheal infections.

To address the growing crisis of antimicrobial resistance in *N. gonorrhoeae* and other pathogens, efforts are underway to develop novel drugs to treat these infections.^13,14,22–24^ The most advanced clinical candidate is gepotidacin (Figure 1), which is a first-in-class triazaacenaphthylene antibacterial.^25–27^ Following priority review,^28^ gepotidacin (Blujepa) was recently approved by the United States Food and Drug Administration (FDA) and the Medicines and Healthcare products Regulatory Agency of the United Kingdom for the treatment of uncomplicated urinary tract infections (caused by uropathogens including *E. coli*)^27^ in adult and adolescent females.^29,30^ Gepotidacin represents the first new class of antibacterials to be approved for the treatment of urinary tract infections in nearly three decades.^29,30^

**Figure 1:**
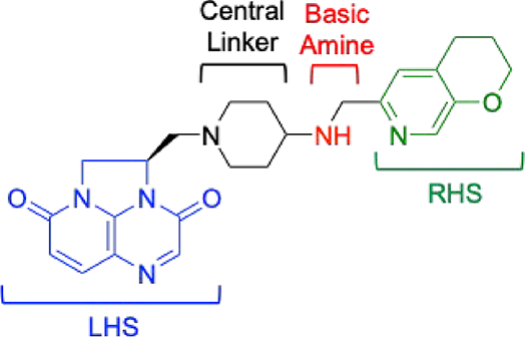
Structure of the triazaacenaphthylene gepotidacin with key pharmacophoric elements shown: left-hand side (LHS, blue) triazaacenaphthylene that pi–pi stacks with two central base pairs of the stretched DNA, central linker (black), basic amine (red) predicted to interact with the aspartic acid (*N. gonorrhoeae* gyrase GyrAD^90^ or topoisomerase IV ParCD^86^, respectively), and right-hand side (RHS, green) that binds in a largely hydrophobic GyrA or ParC pocket that opens up on the dimer interface.

In addition to the approval of gepotidacin for treating patients with urinary tract infections, a recent phase III clinical trial that examined the use of gepotidacin for the treatment of uncomplicated urogenital gonorrhea demonstrated noninferiority to the ceftriaxone plus azithromycin regimen with no new safety concerns.^31,32^ As a result, gepotidacin is currently under priority review by the United States FDA for this second indication^33^ and potentially offers a novel oral treatment option for uncomplicated urogenital gonorrhea.

Gepotidacin targets gyrase and topoisomerase IV but is mechanistically distinct from ciprofloxacin and other fluoro­quinolones. In contrast to the two fluoroquinolone molecules that interact with the enzymes at sites proximal to the two scissile DNA bonds (one drug per scissile bond),^12–14^ a single molecule of gepotidacin binds to gyrase/topoisomerase IV.^22,34,35^ As determined by structural studies with gyrase from *Staphylococcus aureus* ^34^ and *E. coli*,^35^ the left-hand side (Figure 1, LHS) triazaacenaphthylene occupies a DNA pocket on the 2-fold axis of the cleavage complex, positioned midway between the two DNA cleavage sites. The right-hand side (RHS) moiety resides in a pocket on the 2-fold axis between the two GyrA subunits. A key interaction between gyrase/topoisomerase IV and gepotidacin is mediated by an acidic residue (predicted to be GyrA^D90^ and ParC^D86^ in *N. gonorrhoeae* gyrase and topoisomerase IV, respectively), which forms a hydrogen bond with the basic nitrogen of the triazaacenaphthylene.^34,35^ Because gepotidacin interacts with amino acid residues on gyrase and topoisomerase IV that are distinct from those that bind fluoroquinolones, its novel binding motif has the potential to overcome target-mediated fluoroquinolone resistance.^22,34–36^

The effects of gepotidacin on *E. coli* gyrase/topoisomerase IV in purified systems and in cells were reported recently.^36^ In contrast to fluoroquinolones, which kill bacteria primarily through their effects on gyrase in Gram-negative species,^13,14,37^ genetic and biochemical evidence indicate that gepotidacin works in a well-balanced, dual-targeting manner, i.e., it can kill *E. coli* through its effects on either gyrase or topoisomerase IV.^36,38,39^ Genetic studies with *Klebsiella pneumoniae* suggest that gepotidacin also targets this bacterium in a well-balanced, dual-targeting manner.^39^ Consequently, simultaneous gepotidacin target-specific mutations in both gyrase and topoisomerase IV are required to decrease the susceptibility of *E. coli* or *K. pneumoniae* to gepotidacin. This well-balanced dual-targeting confers a significant advantage to gepotidacin over the fluoroquinolones, as it should decrease the possible generation of target-mediated drug resistance.^40^ This characteristic has the potential to extend the clinical lifespan of gepotidacin, at least against *E. coli* and *K. pneumoniae* based infections.

Despite the positive outcome of a clinical trial for the treatment of uncomplicated urogenital gonorrhea with gepotidacin,^25,31,32^ the effects of the triazaacenaphthylene on gyrase and topoisomerase IV from *N. gonorrhoeae* have not yet been reported. Therefore, genetic and biochemical studies were undertaken to determine the targeting of gepotidacin in cultured *N. gonorrhoeae* cells and the in vitro interactions of the drug with purified gyrase and topoisomerase IV from this species. Results indicate that gepotidacin displays well-balanced dual-targeting of gyrase and topoisomerase IV in *N. gonorrhoeae* cells. In support of this finding, gepotidacin inhibited DNA supercoiling and decatenation catalyzed by *N. gonorrhoeae* gyrase and topoisomerase IV, respectively, at similar low micromolar concentrations. Furthermore, as reported with *E. coli*,^36^ gepotidacin induced primarily enzyme-mediated single-stranded DNA breaks and displayed similar potency against both *N. gonorrhoeae* gyrase and topoisomerase IV. Finally, mutation of aspartic acid residues in *N. gonorrhoeae* gyrase (GyrA^D90^) or topoisomerase IV (ParC^D86^), which are predicted to mediate the most important gepotidacin-protein interactions, markedly diminished the activity of gepotidacin against both enzymes. These results provide important insights into the targeting and mechanism of action of gepotidacin.

## Results

### Gepotidacin Exhibits Well-Balanced Dual-Targeting of Gyrase and Topoisomerase IV in *N. gonorrhoeae* Cells

As observed for other Gram-negative bacterial species, gyrase serves as the primary antibacterial target for fluoroquinolones in *N. gonorrhoeae*, while topoisomerase IV serves as a secondary target.^12,13,17,24,37,41^ Because of this unbalanced targeting, a single point mutation in gyrase is often sufficient to allow bacteria to escape the lethal effects of fluoroquinolones. The accumulation of additional mutations in either gyrase or topoisomerase IV can lead to the development of strains that are highly drug-resistant.^14,17,42^

In contrast to fluoroquinolones, drugs that exert their bactericidal effects equally well through gyrase or topoisomerase IV offer an advantage, as target-mediated resistance should require concurrent mutations in both enzymes. Thus, a gyrase/topoisomerase IV-targeted antibacterial that displays well-balanced dual-targeting could potentially be less likely to induce resistance.^40^

The cellular targeting of drugs to bacterial type II topoisomerases can be assessed in two ways. The first is by determining whether resistance mutations first appear in gyrase or topoisomerase IV following the exposure of cells to increasing concentrations of a drug.^43–46^ While this approach is straightforward, it can be confounded by numerous factors, including the levels of drug exposure used to generate resistance, differential enzyme essentiality, and nontarget-based resistance.^43–46^

Although inherently more difficult, the most convincing evidence for drug targeting against the bacterial type II topoisomerases comes from studies that assess drug susceptibility [i.e., minimal inhibitory concentration (MIC)] of isogenic strains that carry resistance mutations in gyrase, topoisomerase IV, or both enzymes.^36,38,39,44,47^ This latter approach was employed to determine the targeting of gepotidacin in *N. gonorrhoeae*.

We utilized a series of isogenic cultured bacterial cell lines based on the FA1090E background strain (Table 1). Strains were wild-type (WT/WT) for both gyrase and topoisomerase IV (FA1090E), contained one of two mutations in GyrA in a background of WT ParC (FA1090E-1, GyrA^A92T^/ParC^WT^ or FA1090E-4, GyrA^D90N^/ParC^WT^), contained a mutation in ParC in a background of WT GyrA (FA1090E-2, GyrA^WT^/ParC^D86N^), or contained mutations in both gyrase and topoisomerase IV (FA1090E-3, GyrA^A92T^/ParC^D86N^ or FA1090E-5, GyrA^D90N/^ParC^D86N^). The gyrase GyrA^A92T^ and topoisomerase IV ParC^D86N^ mutations have been identified in clinical isolates from patients treated with gepotidacin.^48^ The GyrA^D90N^ has not been found clinically but is the equivalent mutation to ParC^D86N^. On the basis of structural studies with *S. aureus* ^34^ and *E. coli* gyrase,^35^ the GyrA^D90^ and ParC^D86^ residues are proposed to be the primary points of contact between gepotidacin and *N. gonorrhoeae* gyrase and topoisomerase IV, respectively.

**Table 1:** Gepotidacin Displays Well-Balanced Dual-Targeting of Gyrase and Topoisomerase IV in Cultured *N. gonorrhoeae* Cells^a^

*N. gonorrhoeae s*train	mutation		gepotidacin MIC (μg/mL)	fold-change
	GyrA	ParC		
**FA1090**	WT	WT	0.5	NA
**FA1090E-1**	A92T	WT	2	4
**FA1090E-2**	WT	D86N	0.5	0
**FA1090E-3**	A92T	D86N	8	16
**FA1090E-4**	D90N	WT	1	2
**FA1090E-5**	D90N	D86N	>64	>128
**FA1090**	WT	WT	0.125	NA
**FA1090-Q1**	S91F/D95G	WT	0.125	0

a The minimum inhibitory concentration (MIC) values of gepotidacin are shown for a collection of isogenic *N. gonorrhoeae* strains. The fold-changes in MIC values from the respective gyrase/topoisomerase IV wild-type (WT/WT) parental strains FA1090 and FA1090E (efflux mutant) are also shown. NA, not applicable.

The susceptibility of these strains to gepotidacin is shown in Table 1, which reports the MIC values for the drug determined using the agar dilution method.^49^ It is notable that gepotidacin is bactericidal against *N. gonorrhoeae* cells^50^ and displayed an MIC value of 0.5 μg/mL against the WT/WT *N. gonorrhoeae* FA1090E (efflux mutant) parental strain. In strains that contained only a single mutation in either gyrase (FA1090-1, GyrA^A92T^ or FA1090E-4, GyrA^D90N^) or topoisomerase IV (FA1090E-2, ParC^D86N^), little or no decrease in gepotidacin susceptibility was observed compared to WT/WT cells (FA1090E). However, when strains that contained both the GyrA^A92T^ and the ParC^D86N^ mutations (FA1090E-3) or both the GyrA^D90N^ and the ParC^D86N^ mutations (FA1090E-5) were examined, the MIC values for gepotidacin rose 16-fold or >128-fold, respectively. These findings provide compelling evidence that gepotidacin can inhibit the growth of *N. gonorrhoeae* cells through its effects on either gyrase or topoisomerase IV. Thus, as was found with *E. coli* ^36^ and *K. pneumoniae*,^39^ gyrase and topoisomerase IV are dual targets for gepotidacin in *N. gonorrhoeae*, and the drug appears to target both enzymes in a well-balanced manner.

The majority (77%) of fluoroquinolone-resistant clinical isolates of *N. gonorrhoeae* that have been analyzed contain a double mutation in the A subunit of gyrase (GyrA^S91F/D95G^).^51^ Although the presence of additional mutations in topoisomerase IV generated in laboratory settings further increases levels of resistance,^37^ they have not been reported in clinical isolates.^51^ Overwhelmingly, target-mediated resistance is the most important form of resistance to fluoroquinolones in *N. gonorrhoeae*. Of the fluoroquinolone-resistant clinical isolates reported to date, only 1.5% lack mutations in gyrase.^51^

To determine whether *N. gonorrhoeae* cells that harbor the most common fluoroquinolone-resistant mutant gyrase (GyrA^S91F/D95G^) retain their susceptibility to gepotidacin, the MIC value of the triazaacenaphthylene against mutant strain FA1090-Q1 was compared to that of parental strain FA1090. As seen in Table 1, the presence of the GyrA^S91F/D95G^ mutation had no effect on the MIC value of gepotidacin (MIC = 0.125 μg/mL). This was compared to a 64-fold reduction in sensitivity toward ciprofloxacin (MIC = 0.25 μg/mL vs 0.004 μg/mL, not shown) in the mutant FA1090-Q1 cells relative to the parental FA1090 line. This finding predicts that gepotidacin should retain its activity against the most common fluoroquinolone-resistant gonorrhea infections.

### Effects of Gepotidacin on the Catalytic Activities of *N. gonorrhoeae* Gyrase and Topoisomerase IV

Compounds that stabilize gyrase or topoisomerase IV-DNA cleavage complexes have two effects on enzyme activity: they decrease the overall catalytic activity and increase the levels of DNA strand breaks generated by the enzymes.^12,13,17,41,52^ Therefore, as a first step toward characterizing the effects of gepotidacin on *N. gonorrhoeae* gyrase and topoisomerase IV, we examined the ability of the drug to inhibit the catalytic activities of these enzymes. This was monitored by a DNA supercoiling assay for gyrase (in which the conversion of relaxed to negatively supercoiled DNA was followed)^53^ and a DNA decatenation assay for topoisomerase IV (in which the conversion of a highly catenated kinetoplast DNA to monomer DNA was followed).^54^

Gepotidacin inhibited DNA supercoiling catalyzed by *N. gonorrhoeae* gyrase with an IC_50_ value (concentration of compound required to reduce enzyme activity by 50%) of 5.1 ± 2.3 μM (Figure 2, left panel, circles) and decatenation catalyzed by topoisomerase IV with an IC_50_ value of 1.8 ± 1.3 μM (right panel, squares). Although gepotidacin was less potent against gyrase than ciprofloxacin (IC_50_ = 0.4 ± 0.02 μM, Figure 2 table), gepotidacin was more potent against topoisomerase IV than the fluoroquinolone (IC_50_ = 13.7 ± 2.4 μM, Figure 2 table). The ∼2.8-fold greater potency for gepotidacin against topoisomerase IV over gyrase is consistent with the balanced dual-targeting of gepotidacin in *N. gonorrhoeae* cells.

**Figure 2:**
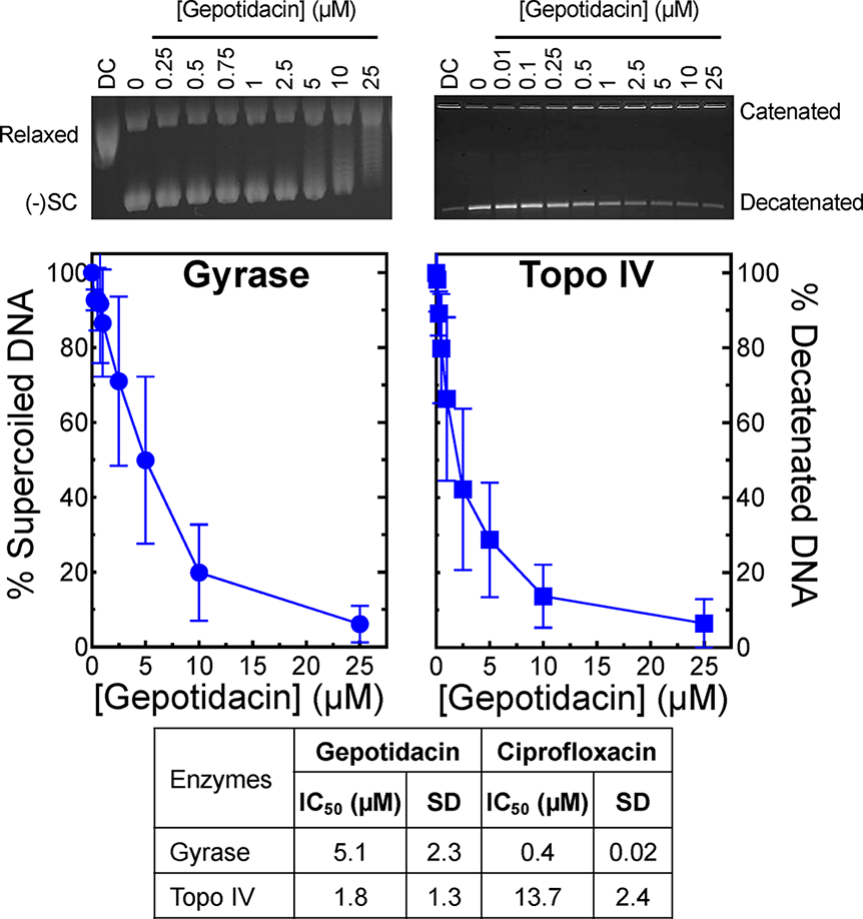
Gepotidacin is a potent inhibitor of WT *N. gonorrhoeae* gyrase-catalyzed DNA supercoiling and WT topoisomerase IV-catalyzed DNA decatenation. The effects of gepotidacin on DNA supercoiling catalyzed by gyrase (left panel, circles) and decatenation catalyzed by topoisomerase IV (right panel, squares) are shown. Error bars represent the standard deviation (SD) of at least three independent experiments. The gels shown at the top are representative supercoiling (left) and decatenation (right) assays with gepotidacin. DC represents the fully relaxed (left) or fully catenated (right) DNA controls. The mobilities of relaxed, negatively supercoiled [(−)­SC], catenated, and decatenated DNA are indicated. IC_50_ values (concentration at which 50% of catalytic activity is inhibited) for gepotidacin are presented in the table at the bottom. IC_50_ values for ciprofloxacin (reported previously)^41^ are shown for comparison.

It should be noted that the supercoiling and decatenation reactions catalyzed by gyrase and topoisomerase IV utilize different DNA substrates (relaxed plasmid pBR322 vs kinetoplast DNA, respectively) and assay conditions. Consequently, although both reactions were optimized for activity, direct comparisons between results from these in vitro assays may not accurately reflect drug targeting in cells. To this point, however, the IC_50_ values for gepotidacin against gyrase-catalyzed DNA supercoiling and topoisomerase IV-catalyzed DNA decatenation are considerably more similar (∼2.8-fold difference) than seen for ciprofloxacin and zoliflodacin, which primarily target gyrase in *N. gonorrhoeae* cells. These latter two drugs are ∼34-fold and >125-fold more potent, respectively, against purified *N. gonorrhoeae* gyrase than topoisomerase IV in supercoiling and decatenation assays.^41,55–58^ Thus, as concluded above, the in vitro inhibition results for gepotidacin are consistent with its well-balanced dual-targeting of gyrase and topoisomerase IV in *N. gonorrhoeae* cells.

### Gepotidacin Induces Single-Stranded DNA Breaks Generated by WT *N. gonorrhoeae* Gyrase and Topoisomerase IV

When fluoroquinolones (two molecules) bind to the bacterial type II topoisomerases, they insert into the cleaved scissile bonds on both strands of the double helix.^12,13,17,41,52,59^ Consequently, fluoroquinolones induce primarily gyrase/topoisomerase IV-mediated double-stranded DNA breaks. In contrast, only a single molecule of gepotidacin binds to the type II enzymes.^34,35^ As a result of its interactions on the 2-fold axis of the cleavage complex, gepotidacin is thought to impose sufficient distortion into the complex after one DNA strand is cleaved to prevent enzyme-mediated cleavage of the second strand.^34^ As a result, gepotidacin induces primarily gyrase/topoisomerase IV-mediated single-stranded DNA breaks and in many cases, suppresses double-stranded scission.^13,14,22,36,60–62^

As seen in Figure 3, gepotidacin enhanced DNA cleavage mediated by *N. gonorrhoeae* gyrase (left panel, circles) and topoisomerase IV (right panel, squares) in a potent manner. Similar to previous results with gepotidacin and related novel bacterial topoisomerase inhibitors (NBTIs) with type II topoisomerases from a variety of bacterial species,^13,14,22,36,60–63^ gepotidacin induced primarily single-stranded DNA breaks (as evidenced by the conversion of negatively supercoiled to nicked molecules, filled symbols) with both enzymes. The CC_50_ (concentration at which 50% maximal DNA cleavage was observed) for gepotidacin against gyrase was 0.9 ± 0.3 μM, with levels of single-stranded DNA breaks maxing at ∼17.1% (adjusted for baseline levels of single-stranded DNA cleavage generated in the absence of gepotidacin) of the initial DNA substrate (left panel, filled circles). Gepotidacin displayed a similar potency against *N. gonorrhoeae* topoisomerase IV, with a CC_50_ value of 0.6 ± 0.2 μM but induced a higher adjusted maximal level of single-stranded DNA scission of ∼42.8% (right panel, filled squares). For both enzymes, gepotidacin was a more potent enhancer of single-stranded DNA cleavage than was reported for double-stranded DNA cleavage by ciprofloxacin (1.3 ± 0.2 μM against gyrase and 7.4 ± 1.9 μM against topoisomerase IV; Figure 3 table).^41^

**Figure 3:**
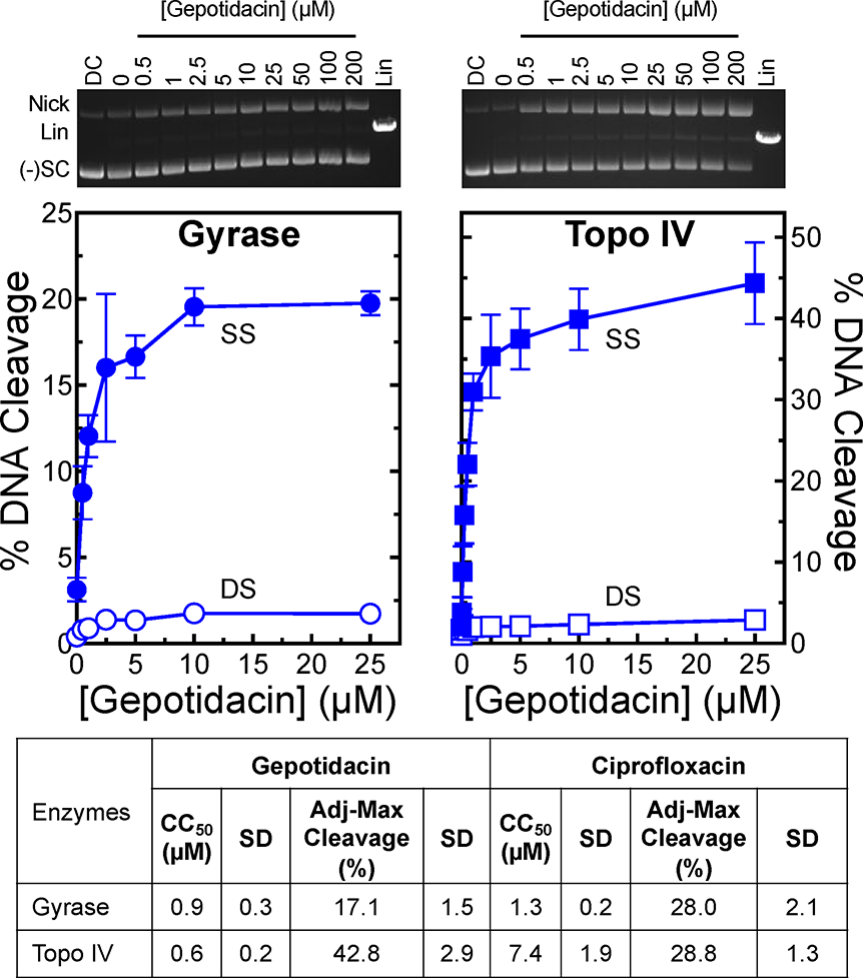
Gepotidacin enhances single-stranded DNA breaks mediated by WT *N. gonorrhoeae* gyrase and topoisomerase IV. The effects of gepotidacin on DNA cleavage mediated by gyrase (left panel, circles) and topoisomerase IV (right panel, squares) are shown. Levels of single-stranded (SS, filled) and double-stranded (DS, open) DNA breaks are shown. Error bars represent the SD of at least three independent experiments. The gels shown at the top are representative DNA cleavage assays with gepotidacin. The mobilities of nicked (Nick or SS), linear (Lin or DS), and negatively supercoiled [(−)­SC] DNA are indicated. The table includes CC_50_ (concentration at which 50% maximal cleavage is induced) and Adj-Max Cleavage (maximal % cleavage induced adjusted for baseline DNA cleavage levels generated in the absence of drugs) values for gepotidacin. Corresponding values for ciprofloxacin (reported previously)^41^ are shown for comparison.

The above DNA cleavage assays were carried out in the absence of ATP. However, to carry out their full catalytic cycles, gyrase and topoisomerase IV require the high energy cofactor ATP.^13–16^ ATP binding dimerizes the N-terminal domains of gyrase/topoisomerase IV, opens the DNA gate, and facilitates passage of the transport DNA helix through the gate.^13–16^ ATP hydrolysis triggers enzyme turnover.^13,14,16,64^ Because bacterial cells are rich in ATP,^65^ DNA cleavage assays were repeated in the presence of the high energy cofactor (Figure 4). ATP had little effect on the potency of gepotidacin against either enzyme or the maximal amount of DNA cleavage induced by the presence of gepotidacin. Furthermore, in the presence of the high energy cofactor, gepotidacin once again induced primarily single-stranded DNA breaks.

**Figure 4:**
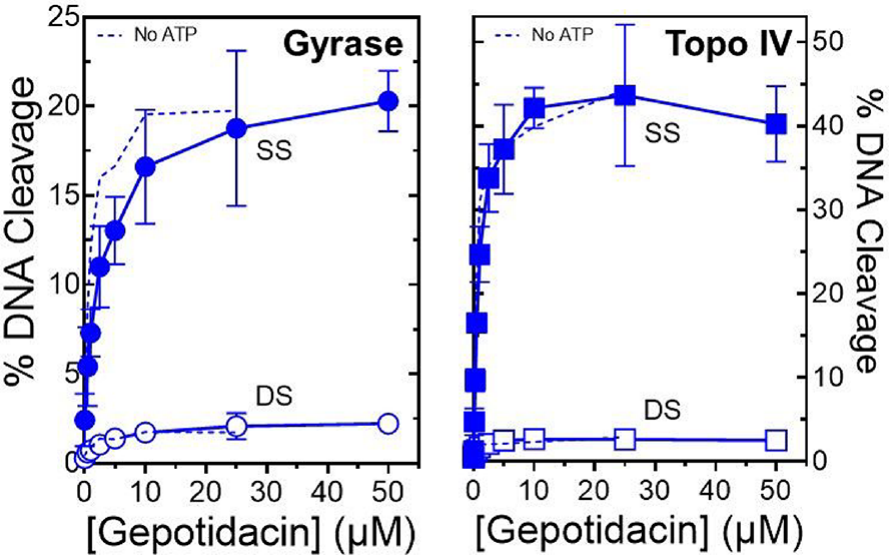
Gepotidacin enhances primarily single-stranded DNA breaks mediated by WT *N. gonorrhoeae* gyrase and topoisomerase IV in the presence of ATP. The effects of ATP on gepotidacin-induced single- (SS, filled) and double-stranded (DS, open) breaks mediated by gyrase (left panel, circles) and topoisomerase IV (right panel, squares) are shown. The dashed lines indicate the effect of gepotidacin on enzyme-mediated cleavage in the absence of ATP (from Figure 3). Error bars represent the SD of at least three independent experiments.

To further explore the ability of gepotidacin to induce single-stranded DNA breaks, two additional experiments were carried out. In the first, gyrase (Figure 5, top) and topoisomerase IV (bottom) were treated with gepotidacin concentrations that were as much as 20-fold higher than saturating concentrations with these enzymes and cleavage assays were carried out for six times longer than normal assays. Under both circumstances, additional enhancement of double-stranded DNA breaks above baseline levels was not observed.

**Figure 5:**
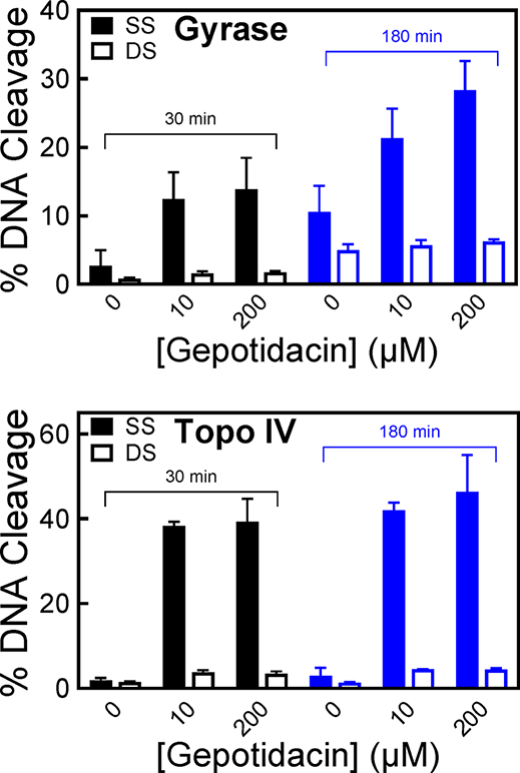
Gepotidacin enhances only single-stranded DNA breaks mediated by WT *N. gonorrhoeae* gyrase and topoisomerase IV. The enhancement of gyrase- (top) or topoisomerase IV-mediated (bottom) single-stranded (SS, filled bar) or double-stranded (DS, open bar) cleavage at 30 min (black) or 180 min (blue) in the presence of 0, 10, or 200 μM gepotidacin, respectively, is shown. Error bars represent the SD of at least three independent experiments.

In the second experiment, Mg^2+^ in the DNA cleavage assays was replaced with Ca^2+^. Type II topoisomerases cleave DNA using a two-metal ion mechanism.^66–68^ In cells, the predominant divalent metal ion is Mg^2+^. Consequently, this is the divalent metal ion that normally is used in DNA cleavage assays. However, when Mg^2+^ is replaced with Ca^2+^, baseline (i.e., drug-free) levels of single- and double-stranded DNA breaks mediated by many bacterial type II topoisomerases increase.^60,68,69^ This allows for a more facile examination of the effects of gepotidacin on the potential suppression of double-stranded breaks by bacterial topoisomerases.

Figure 6 shows the results of DNA cleavage assays performed in the presence of Ca^2+^ for gyrase (left panel) and topoisomerase IV (right panel). There is a clear suppression of double-stranded DNA breaks with topoisomerase IV. Unfortunately, even in the presence of Ca^2+^, levels of double-stranded breaks induced by gyrase were still relatively low, making effects of gepotidacin less obvious. However, it appears that gepotidacin has little effect on double-stranded DNA scission mediated by gyrase.

**Figure 6:**
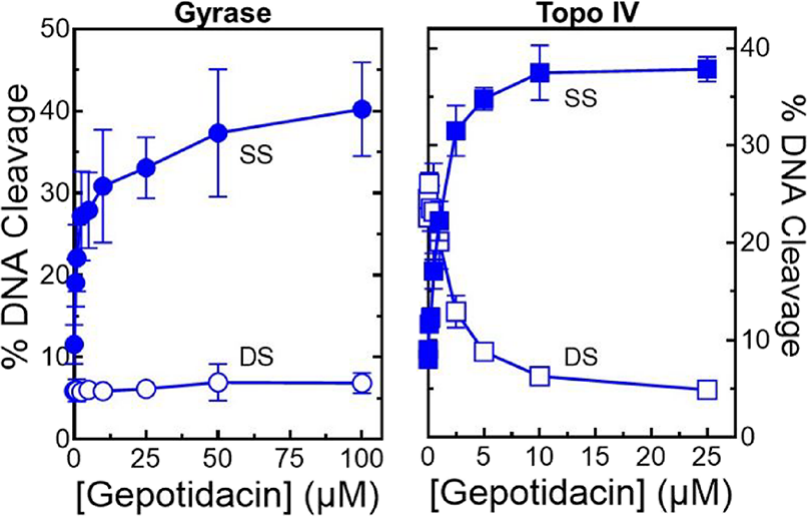
Gepotidacin does not enhance double-stranded DNA breaks mediated by WT *N. gonorrhoeae* gyrase or topoisomerase IV in the presence of Ca^2+^. The effects of Ca^2+^ on gepotidacin-induced single- (SS, filled) and double-stranded (DS, open) DNA breaks mediated by gyrase (left panel, circles) and topoisomerase IV (right panel, squares) are shown. Error bars represent the SD of at least three independent experiments.

### Effects of GyrA and ParC Mutations that Reduce Cellular Susceptibility to Gepotidacin on the Activity of Gepotidacin against *N. gonorrhoeae* Gyrase and Topoisomerase IV

As seen in Table 1, the gyrase GyrA^A92T^ and GyrA^D90N^ mutations and the topoisomerase IV ParC^D86N^ mutation contribute to decreased susceptibility of *N. gonorrhoeae* cells to gepotidacin when combined with a mutation in the opposite enzyme. However, the effects of these individual mutations on the interaction of gepotidacin with *N. gonorrhoeae* gyrase and topoisomerase IV have not been assessed. Therefore, as a first step toward understanding the mechanism that underlies target-mediated resistance to gepotidacin, we examined the ability of the drug to inhibit the catalytic activity (left panel) and enhance the DNA cleavage activity (right panel) of the two gyrase mutants, GyrA^A92T^ and GyrA^D90N^ (Figure 7). Both mutations affected the susceptibility of the enzyme to gepotidacin. The GyrA^A92T^ mutation (black circles) increased the IC_50_ for the inhibition of DNA supercoiling by gepotidacin ∼1.7-fold (from 5.1 ± 2.3 to 8.5 ± 2.1 μM) and decreased the adjusted maximal DNA cleavage by ∼3.5-fold (from 17.1 to 4.9%).

**Figure 7:**
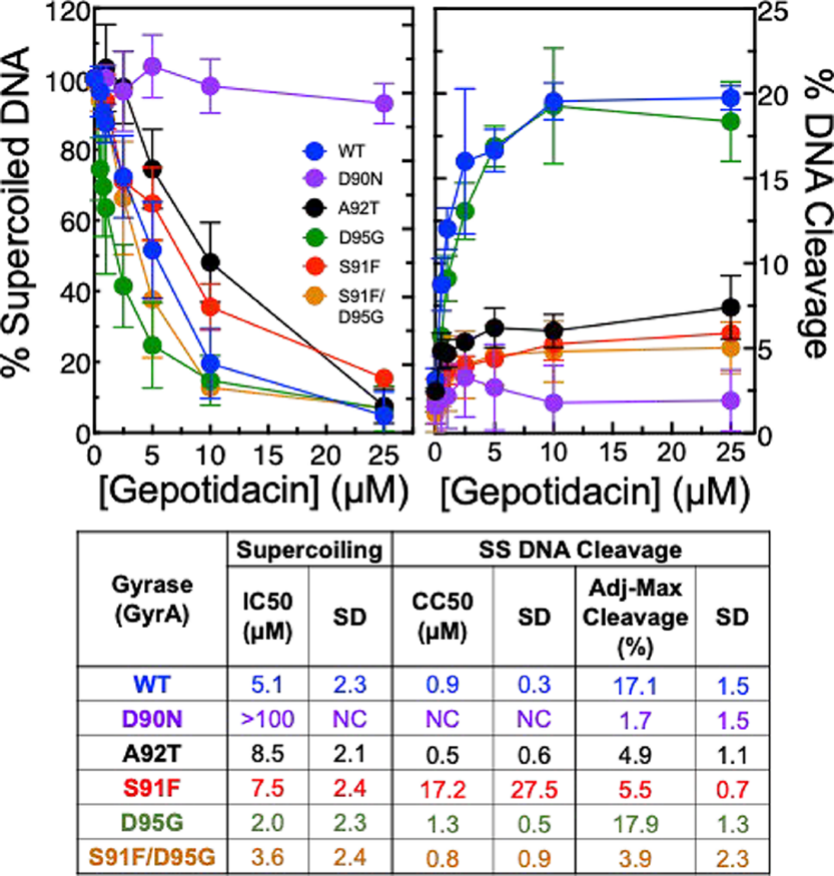
Effects of gepotidacin on DNA supercoiling and DNA cleavage catalyzed by WT *N. gonorrhoeae* gyrase, the mutant GyrA^D90N^ and GyrA^A92T^ enzymes, and the fluoroquinolone-resistant mutant GyrA^S91F^, GyrA^D95G^, and GyrA^S91F/D95G^ enzymes. The effects of gepotidacin on DNA supercoiling catalyzed by WT (blue), mutant GyrA^D90N^ (purple), and GyrA^A92T^ (black) and fluoroquinolone-resistant GyrA^S91F^ (red), GyrA^D95G^ (green), and GyrA^S91F/D95G^ (orange) gyrases are shown in the left panel. Results of DNA cleavage assays with these same enzymes are shown in the right panel. Error bars represent the SD of at least three independent experiments. The table reports IC_50_, CC_50_, and Adj-Max Cleavage values for gepotidacin for comparison between WT and mutant enzymes.

The GyrA^D90N^ mutation had a much more profound effect on the susceptibility of *N. gonorrhoeae* gyrase to gepotidacin. It was not possible to calculate an IC_50_ for gepotidacin for DNA supercoiling by the GyrA^D90N^ mutant enzyme, as very little inhibition was observed (left panel, purple circles). Even at 100 μM gepotidacin, levels of DNA supercoiling with GyrA^D90N^ were ∼93% that of the enzyme in the absence of drug (data not shown). Moreover, essentially no increase in gepotidacin-induced DNA cleavage was observed with this mutant enzyme (right panel, purple circles). The dramatic drop in gepotidacin susceptibility that accompanies the GyrA^D90N^ mutation is consistent with structural studies that indicate that the D90 residue in GyrA is the primary conduit between gyrase and gepotidacin.^34,35^ Furthermore, the greater loss of gepotidacin susceptibility caused by the GyrA^D90N^ mutation as compared to the GyrA^A92T^ mutation is reflected in the cellular studies. Whereas the MIC value for gepotidacin with the FA1090E-3 strain (GyrA^A92T^/ParC^D86N^) was 16-fold higher than that with the wild-type FA1090E (GyrA^WT^/ParC^WT^), the MIC value for the FA1090E-5 strain (GyrA^D90N^/ParC^D86N^) was >128-fold higher (Table 1).

As a follow-up to the in vitro experiments with gyrase, we examined the susceptibility of ParC^D86N^ topoisomerase IV to gepotidacin (Figure 8). As was observed for the equivalent GyrA^D90N^ mutation in gyrase, the ParC^D86N^ mutation dramatically reduced the sensitivity of topoisomerase IV to gepotidacin. Even at gepotidacin concentrations as high as 200 μM, no inhibition of DNA decatenation (left panel, purple squares) was observed and single-stranded DNA cleavage (right panel, purple squares) never rose above 11% (data not shown). These findings support the low susceptibility of *N. gonorrhoeae* cells that harbor the topoisomerase IV ParC^D86N^ mutation in conjunction with a GyrA^A92T^ or GyrA^D90N^ mutation in gyrase (Table 1).

**Figure 8:**
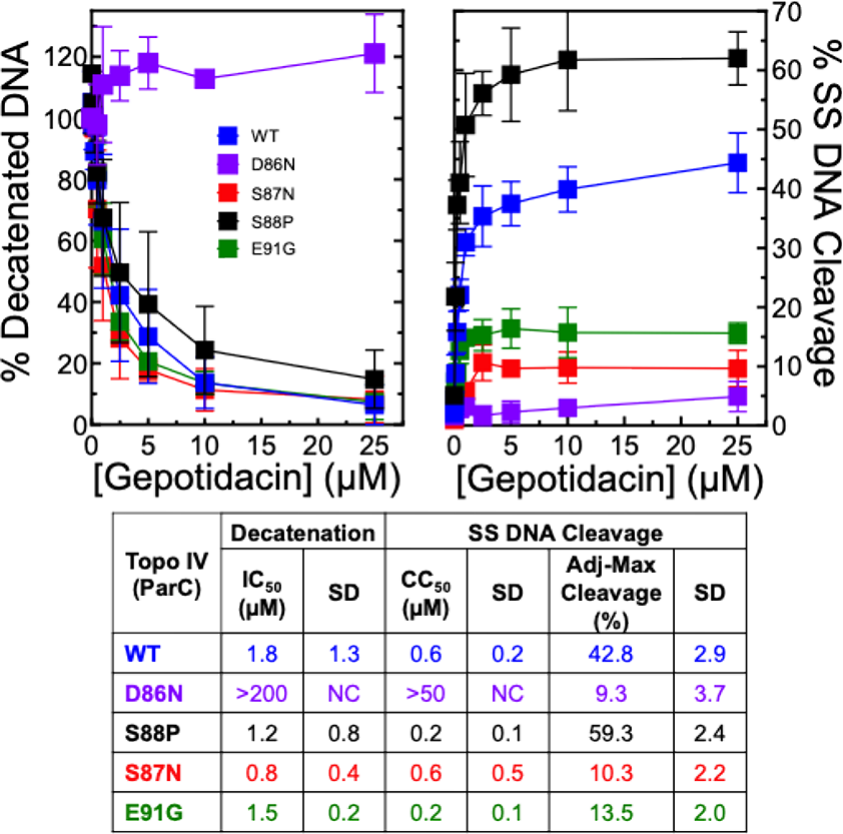
Effects of gepotidacin on DNA decatenation and DNA cleavage catalyzed by WT *N. gonorrhoeae* topoisomerase IV, the mutant ParC^D86N^ and ParC^S88P^ enzymes, and the fluoroquinolone-resistant mutant ParC^S87N^ and ParC^E91G^ enzymes. The effects of gepotidacin on DNA decatenation catalyzed by WT (blue), mutant ParC^D86N^ (purple), and ParC^S88P^ (black), and fluoroquinolone-resistant ParC^S87N^ (red) and ParC^E91G^ (green) topoisomerase IV are shown in the left panel. Results of DNA cleavage assays with these same enzymes are shown in the right panel. Error bars represent the SD of at least three independent experiments. The table reports IC_50_, CC_50_, and Adj-Max Cleavage values for gepotidacin for comparison between WT and mutant enzymes.

As discussed above, the gyrase GyrA^A92T^ mutation reduces susceptibility to gepotidacin in cells and in vitro. However, this amino acid is not conserved and is a serine in topoisomerase IV (ParC^S88^). The ParC^S88P^ mutation has been reported in some clinical strains that also included a second mutation in ParC.^37,70^ Therefore, we examined the potential effects of this mutation on the ability of gepotidacin to inhibit DNA decatenation (Figure 8, left panel, black squares) and induce DNA cleavage (right panel, black squares). The ParC^S88P^ mutation had no effect on gepotidacin susceptibility in topoisomerase IV-catalyzed DNA decatenation assays. Moreover, gepotidacin actually induced ∼40% more single-stranded DNA cleavage with the mutant enzyme as compared to WT topoisomerase IV. Therefore, when considering gepotidacin susceptibility, mutations at this nonconserved residue will have to be considered on an enzyme-by-enzyme basis.

### Effects of Fluoroquinolone-Resistance Mutations on the Susceptibility of *N. gonorrhoeae* Gyrase and Topoisomerase IV to Gepotidacin

Gyrase is the primary cellular target for fluoroquinolones in *N. gonorrhoeae*.^37,71,72^ Mutations most commonly associated with fluoroquinolone resistance occur in a conserved serine residue and an aspartic/glutamic acid residue four amino acids away.^12–14^ In *N. gonorrhoeae* gyrase, these residues are GyrA^S91^ and GyrA^D95^.^41^ GyrA^S91^ and GyrA^D95^ provide the primary conduit for fluoroquinolone binding to *N. gonorrhoeae* gyrase.^41^ These amino acids interact with the drug through water molecules that are coordinated by a noncatalytic divalent metal ion that is chelated by the C3/4 keto acid of the fluoroquinolone skeleton.^59^ Individual GyrA^S91F^ and GyrA^D95G^ mutations have been reported in 19 and 2.5%, respectively, of fluoroquinolone-resistant clinical isolates.^51^ However, the majority (77%) of fluoroquinolone-resistant clinical isolates that have been analyzed contain the mutation in both amino acid residues (GyrA^S91F/D95G^) of gyrase.^51^

To determine the effects of the most common fluoroquinolone-resistance mutations in gyrase^41^ on gepotidacin activity, we examined the susceptibility of gyrase harboring GyrA^S91F^, GyrA^D95G^, or both mutations (GyrA^S91F/D95G^) to gepotidacin (Figure 7). The GyrA^S91F^ mutation reduced susceptibility to gepotidacin somewhat; the IC_50_ for inhibition of DNA supercoiling (left panel, red circles) rose ∼1.5-fold and the adjusted maximal levels of single-stranded DNA cleavage (Adj-Max Cleavage, right panel, red circles) dropped ∼3.1-fold. In contrast, the GyrA^D95G^ mutation had little effect on the susceptibility of gyrase to gepotidacin. The IC_50_ for DNA supercoiling (left panel, green circles) was actually ∼2.6-fold lower with the mutant enzyme compared to WT gyrase and single-stranded DNA cleavage (right panel, green circles) between the two enzymes was nearly indistinguishable.

Gyrase that contained the double GyrA^S91F/D95G^ mutation displayed a slightly enhanced susceptibility in DNA supercoiling assays (left panel, orange circles, IC_50_ = 3.6 ± 2.4 μM as compared to 5.1 ± 2.3 μM for WT) (Figure 7, left panel, orange circles). However, the adjusted maximal levels of single-stranded DNA cleavage (Adj-Max Cleavage, right panel, red circles) dropped ∼4.4-fold to 3.9% (right panel, orange circles).

Topoisomerase IV is a secondary target for fluoroquinolones in cultured *N. gonorrhoeae* cells and mutations in this enzyme contribute to fluoroquinolone resistance when expressed in a background of pre-existing mutations in gyrase.^37,71,72^ Parallel to gyrase, ParC^S87^ and ParC^E91^ provide the primary conduit for fluoroquinolone binding to *N. gonorrhoeae* topoisomerase IV.^41^

Whereas the IC_50_ for the inhibition of DNA decatenation by gepotidacin was ∼2.3-fold lower than WT with the ParC^S87N^ mutation (Figure 8, left panel, red squares), Adj-Max Cleavage levels of single-stranded DNA cleavage induced by gepotidacin dropped ∼4.2-fold (right panel, red squares). Similar results were seen with the ParC^E91G^ mutant topoisomerase IV. The IC_50_ for the inhibition of DNA decatenation by gepotidacin was ∼1.2-fold lower than WT (Figure 8, left panel, green squares) and the Adj-Max Cleavage levels induced by gepotidacin dropped ∼3.2-fold (right panel, green squares).

Taken together, the above results suggest that fluoroquinolone-resistance mutations in gyrase or topoisomerase IV may have variable effects on the susceptibility of gepotidacin at the enzyme level. However, because of the balanced dual-targeting of gepotidacin in *N. gonorrhoeae* cells (Table 1), fluoroquinolone-resistance mutations would have to occur simultaneously in gyrase and topoisomerase IV to influence the susceptibility of infections to gepotidacin. To date, only mutations in gyrase have been reported in fluoroquinolone-resistant clinical isolates.^51^

## Discussion

Gepotidacin is a first-in-class triazaacenaphthylene antibacterial. In a phase III clinical trial for uncomplicated urogenital gonorrhea, gepotidacin met its primary efficacy end point with an overall acceptable safety and tolerability profile compared with intramuscular ceftriaxone plus oral azithromycin.^25,31,32^ Results of the present study indicate that gepotidacin displays well-balanced dual-targeting of gyrase and topoisomerase IV in *N. gonorrhoeae* cells and inhibits bacterial growth equally well through either enzyme. Thus, target-mediated resistance to gepotidacin should only occur when there are concurrent specific mutations in both gyrase and topoisomerase IV. Consequently, at least with regard to target-mediated resistance, the balanced targeting of gepotidacin predicts an extended clinical lifespan of the compound compared to the fluoroquinolone class, which primarily targets gyrase in *N. gonorrhoeae*.^37,71,72^ To this point, results from a large in silico mining of clinical isolates indicated that the concurrence of both GyrA^A92T^ and ParC^D86N^ mutations in *N. gonorrhoeae* was rare.^73^

Balanced dual-targeting of gyrase and topoisomerase IV has been described previously for some fluoroquinolones.^40,44,74,75^ However, it has only been reported for a few members of this drug class and only in the Gram-positive bacteria *Staphylococcus aureus* and *Streptococcus pneumoniae*. Most notably, moxifloxacin, which is widely used clinically, displays balanced dual-targeting in *Streptococcus pneumoniae*.^44,74–76^ Other fluoroquinolones, such as gatifloxacin or garenoxacin, which are used in Japan and parts of Asia, as well as some experimental fluoroquinolones or compounds that failed in clinical trials, also display varying levels of balanced dual-targeting in Gram-positive species.^40,44,74,75^

In contrast to findings with Gram-positive bacteria, balanced dual-targeting of gyrase and topoisomerase IV in Gram-negative bacteria has not been reported for any fluoroquinolone.^14^ Gyrase is always identified as the primary cellular target of fluoroquinolones in Gram-negative species.^14^

In the current study, gepotidacin maintained the ability to inhibit the catalytic activity of gyrase and topoisomerase IV that carried fluoroquinolone-resistance target mutations. Even in the case of the most common double GyrA^S91F/D95G^ mutation, in which levels of gepotidacin-induced single-stranded DNA cleavage were diminished 4.4-fold, gepotidacin displayed full efficacy against an isogenic strain that carried this double mutation (Table 1). It is not known whether the maintenance of sensitivity in cells that harbor the GyrA^S91F/D95G^ mutation is attributable to the potent inhibition of gyrase-catalyzed DNA supercoiling by gepotidacin, the residual actions of the drug against gyrase-mediated DNA cleavage, or the well-balanced dual-targeting of gepotidacin. However, because of the primary targeting of ciprofloxacin to gyrase and the lack of topoisomerase IV mutations in clinical isolates,^51^ our cellular finding leads to the prediction that gepotidacin should maintain high activity against clinically relevant fluoroquinolone-resistant strains of *N. gonorrhoeae*. This aligns with the clinical efficacy demonstrated by gepotidacin against fluoroquinolone-resistant *N*. *gonorrhoeae* isolates from participants in a recent phase III trial.^31^

Recently, a second new class of gyrase/topoisomerase IV-targeted antibacterials, the spiropyrimidinetriones, has emerged.^14,52,77,78^ The most advanced clinical spiropyrimidinetrione, zoliflodacin, also displays activity against *N. gonorrhoeae*,^56^ and results from a phase III clinical trial indicated positive outcomes using zoliflodacin for the treatment of uncomplicated urogenital gonorrhea.^79,80^ Like gepotidacin, zoliflodacin maintains its activity against fluoroquinolone-resistant strains of *N. gonorrhoeae* and appears to be less mutagenic than fluoroquinolones.^55^ However, in contrast to gepotidacin, zoliflodacin displays unbalanced targeting in *N. gonorrhoeae*, and like the fluoroquinolones, preferentially targets gyrase.^55–58^

Finally, the well-balanced dual-targeting of gepotidacin in *N. gonorrhoeae* cells, as well as changes in cellular susceptibility due to mutations in the enzyme targets of this antibacterial, were supported by in vitro studies with purified WT gyrase and topoisomerase IV and mutant enzymes that harbored alterations in amino acid residues predicted to play critical roles in mediating interactions with gepotidacin. Taken together, the present study provides the mechanistic underpinning that connects the actions of gepotidacin against gyrase and topoisomerase IV to its antibacterial activity against *N. gonorrhoeae*.

## Materials and Methods

### Construction of Isogenic *N. gonorrhoeae* Strains with Specific Target Mutations in Gyrase and Topoisomerase IV

A series of isogenic *N. gonorrhoeae* strains was constructed that carried the specific target mutations GyrA^A92T^ or GyrA^D90N^ either alone or in combination with ParC^D86N^ or GyrA^S91F/D95G^ in a ciprofloxacin-susceptible strain, FA1090 (ATCC 700825). An optimized natural transformation protocol using either spot transformation of genomic DNA isolated from *N. gonorrhoeae* strains or DNA amplicons carrying desired alleles generated by PCR site-directed mutagenesis^81,82^ was used to generate the isogenic strains following appropriate selection at various concentrations of gepotidacin or ciprofloxacin.

### Construction of the *N. gonorrhoeae* GyrA^S91F/D95G^ Single Target Mutant Strain

Natural transformation was used to transfer genetic determinants from the donor DNA of a *N. gonorrhoeae* isolate that was ciprofloxacin-resistant (GyrA^S91F/D95G^) and harbored a preexisting ParC^D86N^ substitution^48^ to the ciprofloxacin-susceptible recipient strain FA1090 under selection of ciprofloxacin (0.002 to 2 μg/mL concentration range in GC agar) generating a series of isogenic *N. gonorrhoeae* strains. Ciprofloxacin-resistant transformants were identified carrying the GyrA^S91F/D95G^ single target mutation following DNA sequencing of individual transformants and named FA1090-Q1 (see Table 1 for strain name convention).

### Construction of the *N. gonorrhoeae* ParC^D86N^ Single Target Mutant Strain

To construct the isogenic mutant strains for studying gepotidacin targeting in *N. gonorrhoeae*, genomic DNA was isolated from a baseline *N. gonorrhoeae* clinical trial isolate (GyrA^S91F^, GyrA^D95G^, and ParC^D86N^).^48^ The chromosomal DNA was naturally transformed into *N. gonorrhoeae* FA1090 with selection on gepotidacin at 0.25 μg/mL on GC agar plates (Remel). *N. gonorrhoeae* possesses multiple drug efflux systems (including MtrCDE, FarAB-MtrE, MacAB-MtrE, MATE, and NorM) that play important roles in allowing gonorrhea to escape antibacterials and help this pathogen to evade innate antimicrobial defenses during infection.^83^ Mutations in these efflux genes can result in increased efflux to multiple antibiotics.^84^ Hence, in addition to sequencing the quinolone resistance-determining regions of gyrase and topoisomerase IV, the genes for these efflux genes were also sequenced. DNA sequence analysis of select purified isolates with confirmed reduced susceptibility to gepotidacin revealed that spontaneous mutants that carried a *mtrR*_–79_ single mutation and *mtrR*_–79_/ParC^D86N^ double mutation had been recovered on CG Agar plates containing 0.25 μg/mL of gepotidacin, respectively. mtrR_–79_ is a single T base deletion in the promoter region between *mtrR* (the transcriptional repressor of the MtrCDE efflux pump system) and *mtrCDE*. Parental and isogenic mutant strains generated that carried the efflux mutations mtrR-79 and norM ΔT T6 ^85^ were designated as FA1090E (E for efflux positive). Only the additional GyrA and ParC genotypes are described in Table 1 and in the text. The mutant that additionally expressed ParC^D86N^ was named FA1090E-2.

### Construction of the *N. gonorrhoeae* GyrA^A92T^ ParC^D86N^ Double Target Mutant Strain

To generate the *N. gonorrhoeae* FA1090E-3 strain that expressed both GyrA^A92T^ and ParC^D86N^, GyrA^A92T^ donor DNA generated by site-directed PCR mutagenesis was transformed into *N. gonorrhoeae* FA1090E-2 (ParC^D86N^) recipient cells and selected on GC agar plates supplemented with gepotidacin at 2 μg/mL. DNA sequencing confirmed that the purified isolate acquired GyrA^A92T^. This strain was designated FA1090E-3 (GyrA^A92T^/ParC^D86N^).

### Construction of the *N. gonorrhoeae* GyrA^A92T^ Single Target Mutant Strain

Using the confirmed *N. gonorrhoeae* FA1090E-3 (GyrA^A92T^, ParC^D96N^) double target mutant strain, genomic DNA was prepared and transformed into the FA1090E parent recipient cell followed by selection on GC agar plates containing gepotidacin at 0.5 μg/mL. The purified isolate was confirmed to harbor GyrA^A92T^ as the single target mutation by DNA sequencing. This strain was designated FA1090E-1 (GyrA^A92T^).

### Construction of the *N. gonorrhoeae* GyrA^D90N^ ParC^D86N^ Double Target Mutant Strain

To generate the *N. gonorrhoeae* GyrA^D90N^/ParC^D86N^ double target mutant, GyrA^D90N^ donor DNA was amplified by site-directed PCR mutagenesis, transformed into FA1090E-2 (ParC^D86N^) recipient cells, and selected on GC agar plates containing gepotidacin at 2 μg/mL. DNA sequence analysis of purified isolates confirmed transformation of the GyrA^D90N^ allele into the ParC^D86N^ background. This strain was designated FA1090E-5 (GyrA^D90N^/ParC^D86N^).

### Construction of the *N. gonorrhoeae* GyrA^D90N^ Single Target Mutant Strain

Using the isolate *N. gonorrhoeae* FA1090E-5 (GyrA^D90N^/ParC^D86N^), genomic DNA was prepared and transformed into the parent FA1090E strain with selection on ciprofloxacin at 0.016 μg/mL (ciprofloxacin was chosen because gyrase is the primary cytotoxic target of ciprofloxacin in *N. gonorrhoeae* and ciprofloxacin has selected for low level resistance to ParC^D86N^). DNA sequence analysis of purified isolates confirmed transformation of the GyrA^D90N^ single allele into the ParC^WT^ background. This strain was designated FA1090E-4 (GyrA^D90N^).

### DNA Transformation

All strains were made by utilizing the natural DNA transformation properties of *N. gonorrhoeae* cells.^81^ Genomic DNA was isolated using a DNeasy kit (Qiagen, 69504). PCR primers used for amplification were custom ordered from Integrated DNA Technologies. The quinolone resistance-determining regions of GyrA and ParC were amplified by polymerase chain reaction (PCR) using described primers.^86^ PCR products were amplified using Platinum PCR supermix (Invitrogen, 12532-016) and purified using a PCR cleanup kit (Qiagen, 28106). DNA transformation was performed according to the protocol by Dillard.^82^ Azenta Life Sciences was used to verify successful construction by Sanger DNA sequencing.

### Antimicrobial Susceptibility Testing

All *N. gonorrhoeae* isolates were cultured and grown on GC agar plates or in fastidious broth (Remel, 07664). Minimum inhibitory concentration (MIC) of test compounds was determined by an agar dilution method, the reference testing method for *N. gonorrhoeae* according to the standards of the Clinical and Laboratory Standards Institute and the CDC.^49^ MIC values were determined after incubation at 37 °C for 48 h in 5% CO_2_.

### Materials, DNA, and Enzymes

Gepotidacin mesylate (GSK2140944, GlaxoSmithKline) was stored at −20 °C as 20 mM aliquots in 100% dimethyl sulfoxide (DMSO). All other chemicals were analytical reagent grade.

Negatively supercoiled pBR322 DNA was prepared from *E. coli* using a Plasmid Mega Kit (Qiagen) as described by the manufacturer. Relaxed pBR322 plasmid was generated by treating negatively supercoiled pBR322 with calf thymus topoisomerase I (Invitrogen) in 50 mM Tris-HCl (pH 7.5), 50 mM KCl, 10 mM MgCl_2_, 0.5 mM DTT, 0.1 mM EDTA, and 30 μg/mL bovine serum albumin (BSA) for 45 min at 37 °C followed by heat inactivation of topoisomerase I at 75 °C for 10 min.^87^ Kinetoplast DNA (kDNA) was isolated from *Crithidia fasciculata* as described by Englund.^88^

All proteins were His-tagged. *N. gonorrhoeae* WT gyrase (GyrA, GyrB) and topoisomerase IV (ParC, ParE) subunits as well as mutant GyrA^D90N^, GyrA^A92T^, GyrA^S91F^, and GyrA^S91F/D95G^ gyrase and ParC^D86N^ and ParC^S88P^ topoisomerase IV were prepared as described previously.^22,56,78^ *N. gonorrhoeae* mutant GyrA^D95G^ gyrase and mutant ParC^S87N^ and ParC^E91G^ topoisomerase IV were generated using a QuickChange II XL site-directed mutagenesis kit (Agilent Technologies) with custom primers for the desired mutations. Mutant *N. gonorrhoeae* GyrA and ParC subunits were expressed and purified as described by Ashley et al.^89^ with the following modifications to optimize protein expression and lysis: (1) GyrA^D95G^ was expressed for 2.5 h and ParC^S87N^ and ParC^E91G^ were expressed for 3 h before harvesting, and (2) cells were lysed by sonication using a digital sonifier (Branson). The identities of all constructs were confirmed by DNA sequencing, and all enzymes were stored at −80 °C. In all assays, *N. gonorrhoeae* gyrase or topoisomerase IV was used as a 1:1 GyrA:GyrB or ParC:ParE mixture, respectively, and the stated enzyme concentration reflects that of the holoenzyme (A_2_B_2_).

### Gyrase-Catalyzed DNA Supercoiling Assay

DNA supercoiling assays were based on previously published protocols by Aldred et al.^53^ Assays contained WT GyrB (used at a 1:1 concentration with GyrA), 35 nM (WT GyrA, GyrA^D90N^, GyrA^A92T^, GyrA^D95G^, or GyrA^S91F/D95G^) or 45 nM (GyrA^S91F^) *N. gonorrhoeae* gyrase, 5 nM relaxed pBR322, and 1.5 mM ATP in a total volume of 20 μL of 50 mM Tris–HCl (pH 7.5), 175 mM KGlu, 5 mM MgCl_2_, and 50 μg/mL BSA. Assay mixtures were incubated at 37 °C for 20 min (WT GyrA), 30 min (GyrA^D90N^), 25 min (GyrA^S91F^ or GyrA^S91F/D95G^), 35 min (GyrA^A92T^), or 15 min (GyrA^D95G^), which represent the minimum time required to completely supercoil the DNA in the absence of drug. Reactions were stopped by the addition of 3 μL of a mixture of 0.77% SDS and 77.5 mM Na_2_EDTA. Samples were mixed with 2 μL of loading dye [60% sucrose, 10 mM Tris–HCl (pH 7.9), 0.5% bromophenol blue, and 0.5% xylene cyanol FF] and incubated at 45 °C for 2 min before being subjected to electrophoresis on 1% agarose gels in 100 mM Tris-borate (pH 8.3) and 2 mM EDTA. Gels were stained with 1 μg/mL ethidium bromide for 20 min, then destained with distilled water for 10 min. DNA bands were visualized with medium-range ultraviolet light and quantified using an Alpha Innotech digital imaging system (Protein Simple). DNA supercoiling was monitored by the conversion of relaxed to supercoiled plasmid. IC_50_ values (the concentration of drug required to inhibit enzyme activity by 50%) were calculated on GraphPad Prism Version 10.0.3 using the analysis ″[inhibitor] vs response – Variable slope (four parameters) nonlinear least-squares fit″ analysis with 95% confidence intervals using the equation:

$$Y=\mathrm{Bottom}+\frac{\left(\mathrm{Top}-\mathrm{Bottom}\right)}{1+{\left(\frac{{\mathrm{IC}}_{50}}{x}\right)}^{\mathrm{Hill}\;\mathrm{slope}}}$$

and represent the concentration of gepotidacin that decreased supercoiling activity by 50%.

### Topoisomerase IV-Catalyzed DNA Decatenation Assay

DNA decatenation assays were based on previously published protocols by Anderson et al.^90^ and Aldred et al.^54^ Assays contained WT ParE (used at a 1:1 concentration with ParC), 7.5 nM WT ParC, 15 nM mutant *N. gonorrhoeae* (ParC^D86N^, ParC^S87N^, or ParC^S88P^) or 35 nM (ParC^E91G^) topoisomerase IV, 5 nM kDNA, and 1 mM ATP in 20 μL of 40 mM HEPES–KOH (pH 7.6), 25 mM NaCl, 100 mM KGlu, and 10 mM Mg­(OAc)_2_. Assay mixtures were incubated at 37 °C for 15 min (WT ParC or ParC^S88P^), 20 min (ParC^D86N^), 25 min (ParC^S87N^), or 60 min (ParC^E91G^), which represent the minimum time required to completely decatenate the kDNA in the absence of drug. Reactions were stopped, subjected to electrophoresis, and visualized as described for gyrase-catalyzed DNA supercoiling. IC_50_ values were calculated on GraphPad Prism Version 10.0.3 using a nonlinear regression analysis with 95% confidence intervals as described in the previous section.

### DNA Cleavage Assay

DNA cleavage reactions were performed according to the procedure of Aldred et al.^54^ Reactions were performed in the absence or presence of increasing concentrations of gepotidacin. Unless stated otherwise, assay mixtures contained 10 nM pBR322, WT GyrB (used at a 1:1 concentration with GyrA), and 100 nM WT GyrA, GyrA^D90N^, GyrA^A92T^, GyrA^S91F^, GyrA^D95G^, or GyrA^S91F/D95G^ *N. gonorrhoeae* gyrase or WT ParE (used at a 1:1 concentration with ParC), 100 nM WT ParC, 200 nM ParC^D86N^, ParC^S88P^, or ParC^S87N^, or 150 nM ParC^E91G^ *N. gonorrhoeae* topoisomerase IV in a total volume of 20 μL of 40 mM Tris–HCl (pH 7.9), 50 mM NaCl, 2.5% (w/v) glycerol, and 10 mM MgCl_2_. Some experiments replaced MgCl_2_ with an equivalent concentration of CaCl_2_ (10 mM), or 1.5 mM ATP was added to assay mixtures.

Reactions were incubated at 37 °C for 30 min with WT and mutant (GyrA^D90N^, GyrA^A92T^, GyrA^S91F^, GyrA^D95G^, or GyrA^S91F/D95G^) *N. gonorrhoeae* gyrase, 10 min with WT *N. gonorrhoeae* topoisomerase IV, and 20 min with mutant (ParC^D86N^, ParC^S88P^, ParC^S87N^, or ParC^E91G^) *N. gonorrhoeae* topoisomerase IV. Enzyme-DNA cleavage complexes were trapped by adding 2 μL of 5% SDS followed by 2 μL of 250 mM EDTA (pH 8.0). Proteinase K was added (2 μL of a 0.8 mg/mL solution), and reaction mixtures were incubated at 45 °C for 30 min to digest the enzyme. Samples were mixed with 2 μL of loading buffer and heated at 45 °C for 2 min prior to electrophoresis in 1% agarose gels in 40 mM Tris-acetate (pH 8.3), and 2 mM EDTA containing 0.5 μg/mL ethidium bromide. DNA bands were visualized by midrange ultraviolet light and quantified using an Alpha Innotech digital imaging system (Protein Simple). Single-stranded DNA cleavage was monitored by the conversion of negatively supercoiled to nicked plasmid molecules. CC_50_ values were calculated using the analysis ″[agonist] vs response – Variable slope (four parameters) nonlinear least-squares fit″ with 95% confidence intervals provided by GraphPad Prism 10.0.3 using the equation:

$$Y=\mathrm{Bottom}+\frac{\left(x^{\mathrm{Hill}\;\mathrm{slope}})(\mathrm{Top}-\mathrm{Bottom}\right)}{x^{\mathrm{Hill}\;\mathrm{slope}}+{\mathrm{EC}}_{50}^{\mathrm{Hill}\;\mathrm{slope}}}$$

and represent the concentration of gepotidacin that induced 50% of the adjusted maximal DNA cleavage.

## Notes

*N. gonorrhoeae* gyrase, GyrA: UniProtKB P48371, GyrB: UniProtKB P22118; *N. gonorrhoeae* topoisomerase IV, ParC: UniProtKB P48374, ParE: UniProtKB A0A8D9YA86.

## ⊥

Department of Microbiology, Genetics, and Immunology, Michigan State University, East Lansing, Michigan 48824, United States
